# The Inflammatory Side of Iatrogenic Cerebral Amyloid Angiopathy: Rethinking Therapeutic Opportunities

**DOI:** 10.3390/brainsci16010075

**Published:** 2026-01-06

**Authors:** Mattia Losa, Andrea Donniaquio, Ilaria Gandoglia, Federico Massa, Fabio Gotta, Luca Sofia, Lorenzo Gualco, Enrico Peira, Andrea Chincarini, Luca Roccatagliata, Fabrizio Piazza, Massimo Del Sette, Matteo Pardini

**Affiliations:** 1 Department of Neuroscience, Rehabilitation, Ophthalmology, Genetics, Maternal and Child Health (DINOGMI), University of Genoa, 16132 Genoa, Italy; mattia.losa@outlook.it (M.L.); fedemassa88@gmail.com (F.M.); fabio.gotta@hsanmartino.it (F.G.); lorenzo.ovada@gmail.com (L.G.); 2 E.O. Ospedali Galliera, 16128 Genoa, Italy; andrea.donniaquio@gmail.com; 3 IRCCS Ospedale Policlinico San Martino, 16132 Genoa, Italy; ilaria.gandoglia@hsanmartino.it (I.G.); massimo.delsette@hsanmartino.it (M.D.S.); 4 Department of Health Science (DISSAL), University of Genoa, 16132 Genoa, Italy; luca.sofia97@gmail.com; 5 Neuroradiology Unit, Azienda Ospedaliero-Universitaria Santi Antonio e Biagio e Cesare Arrigo, 15121 Alessandria, Italy; 6 Genoa Section, National Institute of Nuclear Physics (INFN), 16146 Genoa, Italy; enrico.peira@ge.infn.it (E.P.); andrea.chincarini@ge.infn.it (A.C.); 7 Neuroradiology, Department of Health Sciences (DISSAL), University of Genoa, 16132 Genoa, Italy; luca.roccatagliata@unige.it; 8 CAA and AD Translational Research and Biomarkers Laboratory, School of Medicine and Surgery, University of Milano-Bicocca, 20126 Monza, Italy; fabrizio.piazza@unimib.it

**Keywords:** cerebral amyloid angiopathy, iatrogenic CAA, CAA-ri, neuroinflammation, hemorrhagic risk

## Abstract

Background: Iatrogenic cerebral amyloid angiopathy (iCAA) is a rare form of CAA occurring decades after neurosurgical procedures involving cadaveric dural grafts. While typically associated with recurrent lobar intracerebral hemorrhages, recent reports suggest a possible overlap with CAA-related inflammation (CAAri). We report a case of iCAA with features indicative of active neuroinflammation that demonstrated a positive response to immunosuppressive therapy. Methods: Over a 12-year natural history, the patient underwent a comprehensive work-up, including serial clinical assessments, brain MRIs, core CSF biomarker analysis, amyloid PET imaging, and next-generation sequencing panel testing. Results: Previous clinical charts confirmed the use of cadaveric graft (Lyodura) in a neurosurgical intervention thirty years before. During hospitalization for seizures, brain MRI revealed, along with a severe form of CAA, an area of vasogenic edema. Given the suspicion of an active inflammatory process, corticosteroid and subsequent methotrexate maintenance therapy were introduced, leading to clinical and radiological improvement. Over 30 months of follow-up, the patient has remained clinically and radiologically stable, with no new hemorrhagic or inflammatory events. Conclusions: This case highlights the potential interplay between iCAA and neuroinflammation. The absence of new hemorrhages following immunosuppression suggests a possible disease-modifying effect, warranting further investigation into the role of neuroinflammation in iCAA and its therapeutic implications.

## 1. Introduction

An increasing number of Cerebral Amyloid Angiopathy (CAA) cases following neurosurgical procedures have been reported, a condition referred to as iatrogenic CAA (iCAA) [1]. In these cases, a prion-like mechanism involving cadaveric dural grafts drives the pathogenetic process [2]. Suggested diagnostic criteria for iCAA have been published, including, for the first time, amyloid biomarkers to support CAA diagnosis [1].

CAA can be complicated by an autoinflammatory response against beta-amyloid, producing a typical clinical-radiologic syndrome, known as CAA-related inflammation (CAAri) [3]. Notably, several reports and a recently published multicenter study identified a conspicuous overlap between iCAA and CAAri, with 27.4% of iCAA cases exhibiting MRI inflammatory abnormalities during the natural course [2,4]. These resemble amyloid-related imaging abnormalities (ARIA) of patients exposed to anti-amyloid therapy (AAT) drugs [5]. Nevertheless, the clinical significance of these findings in iCAA remains unclear. Neuroinflammation may potentially have a protective effect in subjects with a highly reactive immune system or be a deleterious pathophysiological mechanism, enhancing blood-brain-barrier (BBB) leakage and vessel rupture, thus representing a promising therapeutic target [6].

Here, we present a well-characterized case of iCAA with an overlapping inflammatory flare-up, which benefited from immunosuppressive therapy.

## 2. Materials and Methods

The patient has been clinically followed at the IRCCS Ospedale Policlinico San Martino (Genoa, Italy) from 2017 to 2025, with serial MRI acquired with the same protocol from 2022 to 2025 (Supplementary Materials). We retrospectively collected the clinical history from previous records. Amyloid PET was performed using [^18^F]florbetapir, and images were analyzed using DOlab Research Version 0.5.5 (Dorian Technologies S.r.l.) [7,8]. Core CSF biomarkers (Aβ42, Aβ40, p-Tau181, total-Tau) and Next-Generation Sequencing (NGS) gene panel were performed to support diagnosis and exclude hereditary CAA (Supplementary Materials). The manuscript was prepared in compliance with the CAse REport (CARE) guidelines.

## 3. Case Report

The patient had no cardiovascular risk factors, family history of neurological disorders (including stroke and dementia), or history of head trauma. In 1984, he underwent surgical decompression of a Chiari malformation. In 2013, at the age of 56, the patient was evaluated for post-exercise headache, with evidence of an acute right temporal lobar ICH and a chronic ICH in the contralateral temporal lobe. Angiographic investigation was negative for macrovascular abnormalities. In 2015, following an intense headache episode, he experienced recurrent, short-lasting, left-sided sensory symptoms, which were interpreted as focal seizures (although possibly compatible with transient focal neurological episodes, TFNE). In the following two years, three novel ICHs occurred in the left and right frontal lobes, with residual behavioral disturbances (i.e., irritability, apathy).

**Figure 1:**
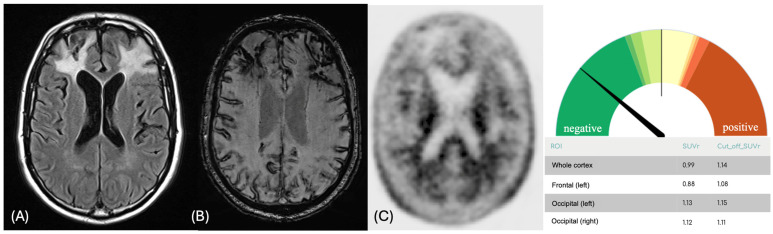
Brain MRI and Amyloid PET of the patient. (**A**) FLAIR and (**B**) SWI sequences of the patient in 2017, which showed extensive bifrontal ICHs with massive disseminated cortical superficial siderosis. (**C**) Amyloid PET tracer uptake ([^18^F]Florbetapir) was reduced in the frontal lobes due to previous lobar ICH. The exam was visually reported as negative for amyloid accumulation, but the semi-quantitative analysis showed borderline SUVr values in the occipital lobes. The Gauge plot provides a graphical representation of the semi-quantitative whole-brain tracer uptake. Semi-quantitative analysis was performed using DOlab Research Version (Dorian Technologies S.r.l.), with regional SUVr values normalized to the cerebellar parenchyma (whole brain SUVr threshold for positivity: 1.14). Legend: ICH = intracerebral hemorrhage; PET = Positron Emission Tomography; SUVr = Standardized Uptake Value ratio.

In 2017, a brain MRI performed in our center confirmed an extensive cortical superficial siderosis, along with multiple lobar ICHs and microbleeds (Figure 1). An amyloid PET scan in the same year was visually reported as negative for amyloid accumulation. Semi-quantitative analysis, normalized to cerebellar parenchyma, revealed a global [^18^F]Florbetapir SUVr of 0.99, which is below the commonly accepted positivity threshold for Alzheimer’s disease (AD − VN < 1.14). However, regional analysis showed borderline SUVr values in the occipital cortex bilaterally (Left Occipital SUVr = 1.13, VN < 1.15; Right Occipital SUVr = 1.12, VN < 1.11—Figure 1 and Supplementary Materials). The CSF examination performed in 2017 revealed reduced levels of Aβ42 (363 pg/mL; VN > 600 pg/mL) and increased levels of p-tau181 (63.7 pg/mL; VN < 56.5 pg/mL) and t-Tau (473 pg/mL; VN < 404 pg/mL—Aβ40 was not assessed as it was not a routine test in our lab at the time). Genetic analysis revealed ApoE ɛ3 homozygosity, while an NGS panel for monogenic cerebral small vessel disease was unremarkable (Supplementary Materials). Based on these findings, a diagnosis of early-onset probable CAA associated with AD was made [9]. The patient experienced other ICHs in 2019 and 2021, with an annual incidence from onset of 0.67 events/year.

**Figure 2:**
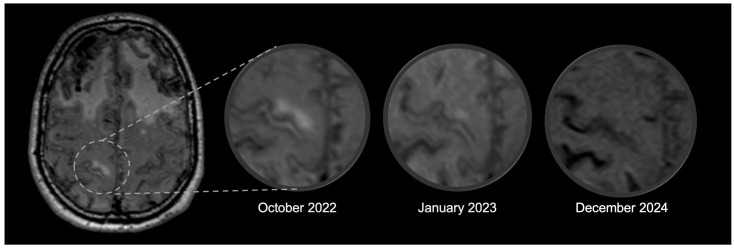
Spontaneous area of vasogenic edema (ARIA-E) in an iCAA patient. The alterations in the right precentral gyrus visible in FLAIR gradually resolved following immunosuppression. Legend: ARIA = Amyloid-related Imaging Abnormalities; iCAA = iatrogenic Cerebral Amyloid Angiopathy; E = edema/effusion.

In October 2022, the patient was admitted to our Neurology Unit for suspected generalized epileptic seizures and psychomotor retardation. During the hospitalization, levetiracetam was initiated, with regression of the episodes, and owing to the patient’s history, the operation notes from three decades before were retrieved, with evidence of cadaveric dura mater graft (Lyodura), confirming the diagnosis of probable iCAA [1]. Brain MRI performed at this time point revealed a hyperintense area at the level of the right precentral gyrus, suggestive of vasogenic edema (Figure 2), with associated diffuse sulcal hyperintensities in the FLAIR sequence and without clear contrast enhancement in the post-gadolinium T1 sequences. These findings were absent in the previous MRI. A lumbar puncture was repeated, which showed mild hyperproteinorrachia without pleocytosis or intrathecal synthesis, and confirmed low amyloid markers (Aꞵ42 332 pg/mL, −8.5% compared to 2017; Aꞵ40 6723 pg/mL, Aꞵ42/40 ratio 0.049) and increased Tau proteins (total-Tau 661 pg/mL, +39.7%; p-Tau181 78 pg/mL, +22.4%). Autoimmune panel, neurotropic virus, and cytological examination in CSF were negative. Cognitive deficits were assessed through the Mini-Mental State Examination (MMSE), with a result of 18/30. In the suspicion of an inflammatory flare-up [3], intravenous methylprednisolone (500 mg for 5 days) was administered, followed by slow oral tapering. In the subsequent few days, the patient improved in terms of psychomotor slowing and cognitive performance (MMSE 27/30 at the follow-up evaluation after one month). Brain MRI performed after three months showed a reduction in the vasogenic edema (Figure 2), with complete resolution in the subsequent scans (monitored after 6 months, and then annually). A maintenance therapy with methotrexate 7.5 mg/week was introduced as a steroid-sparing treatment, selected based on our clinical experience.

**Figure 3:**
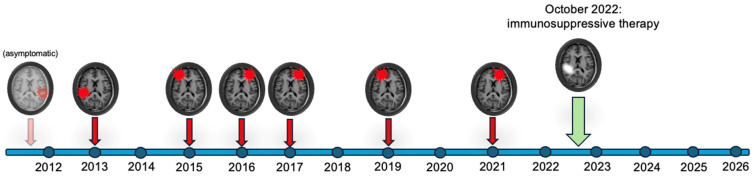
Spontaneous intracerebral hemorrhages (shown in red) and (known) spontaneous ARIA-E (shown in white) developed in the patient during his lifetime. Legend: ARIA = Amyloid-related Imaging Abnormalities; E = edema/effusion.

No further ICHs or inflammatory changes have been reported since discharge in the follow-up brain MRIs (Figure 3). At the 30-month follow-up visit, the patient was cognitively stable (MMSE 26/30), and the treatment was well tolerated, with no adverse effects or laboratory abnormalities reported at regular biochemical monitoring. The caregiver confirmed both cognitive and functional stability, despite reporting persistent and slight worsening of the behavioral disturbances.

## 4. Discussion

This case highlights the overlap between iCAA and CAA-ri, how neuroinflammation may represent a therapeutic opportunity, and the possible pitfalls of amyloid biomarkers in this clinical context.

The spectrum of CAAri is currently expanding, reflecting the identification of patients with radiological findings that do not fully meet current in vivo diagnostic criteria and the observation of spontaneous ARIA-E in AD patients enrolled in AAT trials [5,10,11]. In our iCAA case, the presence of cortical-subcortical vasogenic edema and sulcal hyperintensities, together with the marked clinical and radiologic improvement following steroid therapy, strongly points to the contribution of an immune-mediated process. A growing number of reports support a role of neuroinflammation also in non-inflammatory CAA, particularly in the so-called vascular remodeling [6,12], and suggest a potential influence on CAA-related hemorrhagic risk [13]. Although data remain limited, the absence of hemorrhagic events after immunosuppression in our case raises the possibility of a disease-modifying effect of immunosuppressive therapy. This, however, remains speculative and warrants further investigation in larger cohorts.

Amyloid PET-CSF mismatch has been previously described in AD [14] and iCAA [15]. In this case, the incongruity may largely be explained by the presence of extensive hemorrhagic sequelae, which likely contributed to the reduced frontal and temporal tracer uptake observed [15]. However, regional semi-quantitative analysis revealed evidence of occipital amyloid deposition, aligning with the pathological CSF despite the negative visual and global PET assessment. An additional contributing factor of these findings may be the inflammatory process, which has been associated with a reduction in amyloid tracer uptake [16] and vascular amyloid-beta clearance [12]. This case highlights a potential caveat of amyloid PET in CAA, which should be interpreted with caution, considering the different pattern and global burden of amyloid-beta accumulation compared to AD [17]. CSF biomarkers may therefore ensure a higher diagnostic accuracy in this context compared to amyloid PET, but future head-to-head studies should be performed to establish a clear diagnostic performance comparison. Notably, this patient also exhibited an A+T+ CSF biomarker profile [9] with reduced Aꞵ40 level, supporting both the biological diagnosis of CAA [18] and the presence of comorbid (iatrogenic) AD.

In summary, this case puts neuroinflammation in iCAA under the spotlight, raising questions about the significance of spontaneous ARIA-like events in these patients, which deserve a deeper exploration. Further research is warranted for identifying reliable markers to track in vivo inflammatory activity in CAA and to confirm the potential opportunity of treating neuroinflammation in iatrogenic (and sporadic) CAA.

## Data Availability

The data underlying this study include sensitive patient information and cannot be made publicly available due to ethical and legal constraints. Anonymized data may be shared by the corresponding author upon reasonable request.

## Supplementary Materials

The following supporting information can be downloaded at: https://www.mdpi.com/article/10.3390/brainsci16010075/s1, (1) MRI protocol; (2) Lumbar puncture and CSF analysis; (3) Next-Generation Sequencing gene panel. Supplementary Figure S1—Analysis of amyloid PET using DOlab Research Version (Dorian Technologies S.r.l.), which provides different semi-quantitative values (SUVr, ELBA, and TDr) in various regions of interest [7,8].

## Abbreviations

The following abbreviations are used in this manuscript:

AAT	Anti-Amyloid Therapies
AD	Alzheimer’s Disease
ARIA	Amyloid-Related Imaging Abnormalities
CAA	Cerebral Amyloid Angiopathy
CAA-ri	Cerebral Amyloid Angiopathy-related inflammation
CSF	Cerebrospinal Fluid
iCAA	Iatrogenic Cerebral Amyloid Angiopathy
ICH	Intracerebral Hemorrhage
MMSE	Mini-Mental State Examination
MRI	Magnetic Resonance Imaging
NGS	Next Generation Sequencing
PET	Positron Emission Tomography
TFNE	Transient Focal Neurological Episodes
