# Supplementary material for: The Inflammatory Side of Iatrogenic Cerebral Amyloid Angiopathy: Rethinking Therapeutic Opportunities
Source: Brain Sci. 2026 Jan 6;16(1):75. doi: 10.3390/brainsci16010075 (PMC12838659; doi:10.3390/brainsci16010075)
Supplement: Supplementary file 1 [file brainsci-16-00075-s001.zip › brainsci-4035915-supplementary.pdf]

Supplementary Material

1) MRI Protocol (from 2022 to 2025)

MRI was performed on a clinical 3T MR scanner (MAGNETOM Prisma Siemens Healthineers, Erlangen, Germany), using a 64-channel multiarray receiver coil. Each scan included a 3D T2-FLAIR sequence with the following parameters: TR = 4500 ms, TE = 383 ms, TI = 1800 ms, FA = 120°; a 3D T1 MPRAGE with the following parameters: TR = 2300 ms, TE = 2.98 ms, TI = 919 ms, FA = 9°; a 2D T2* GRE with the following parameters: TR = 886 ms, TE = 20 ms, FA = 20°.

2) Lumbar puncture and CSF analysis

Following standard operating procedures, CSF samples (6 to 8 mL) were obtained via lumbar puncture at the L3-L4 or L4-L5 interspace in the early morning[29]. The CSF was collected using sterile polypropylene tubes, then centrifuged at 4000 g for 10 minutes at 4°C. To ensure the long-term stability of proteins, the resulting aliquots were stored in polypropylene tubes at -80°C until analysis. We utilized the Lumipulse G600 II® fully automated chemiluminescent enzyme immunoassay system (Fujirebio Europe, Gent, Belgium) to measure core AD-related CSF biomarkers (Aβ42, Aβ40, p-Tau181, and t-Tau). Assay cartridge datasheet cut-offs were also verified in our centers: 600 pg/mL for Aβ42; 0.069 for Aβ42/40 ratio, 404 pg/mL for total Tau; 56.5 pg/mL for pTau.

3) Next-Generation Sequencing gene panel

Blood samples from the proband were collected, and genomic DNA was extracted from peripheral blood leukocytes using standard methods. Next-Generation Sequencing (NGS) analyses were conducted with AmpliSeq custom gene panels. A total of 13 genes (*APP*, *COL4A1*, *COL4A2*, *COLGALT1*, *CST3*, *CTSA*, *FOXC1*, *FOXF2*, *GLA*, *HTRA1*, *ITM2B*, *NOTCH3*, *TREX1*) were analysed on an Ion GeneStudio S5 System sequencer (Thermo Fisher, Waltham, Massachusetts, USA).

Supplementary figure – Analysis of amyloid PET using DOlab Research Version (Dorian Technologies S.r.l.), which provides different semi-quantitative values (SUVr, ELBA and TDr) in various regions of interest[1,2].

Legend: L=Left; R=right; ROI=Regions Of Interest; SUVr= Standardized Uptake Value Ratio; TDR=Time-delayed ratio, TDr

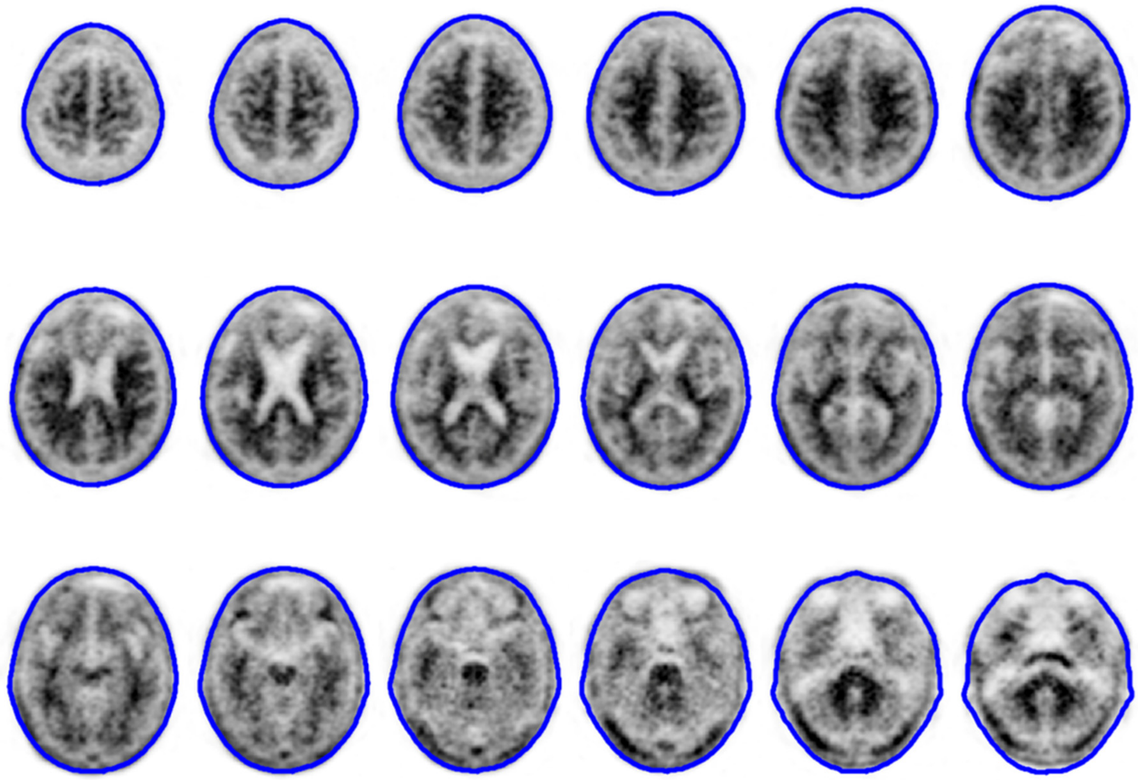

ROI	SUVr	Cut_off_SUVr	ELBA	Cut_off_ELBA	TDr	Cut_off_TDr
Whole-brain	0.99	1.14	0.72	0.89	0.52	0.61
L_Frontal	0.88	1.08	0.63	0.85	0.54	0.61
L_Occipital	1.13	1.15	0.83	0.83	0.51	0.58
L_Posterior_Parietal	1.11	1.15	0.83	0.82	0.53	0.60
L_Lateral_Temporal	1.05	1.13	0.72	0.80	0.50	0.58
L_Precuneus_+_Post_Cingulate	1.06	1.10	0.81	0.85	0.50	0.61
R_Frontal	0.85	1.11	0.58	0.85	0.52	0.62
R_Occipital	1.12	1.11	0.82	0.81	0.52	0.57
R_Posterior_Parietal	1.10	1.13	0.77	0.85	0.53	0.61
R_Lateral_Temporal	1.01	1.10	0.67	0.81	0.52	0.59
R_Precuneus_+_Post_Cingulate	1.09	1.12	0.79	0.84	0.49	0.62
